# Case report: Ensitrelvir for treatment of persistent COVID-19 in lymphoma patients: a report of two cases

**DOI:** 10.3389/fimmu.2024.1287300

**Published:** 2024-01-25

**Authors:** Chiho Furuya, Hajime Yasuda, Makoto Hiki, Shuichi Shirane, Tomohito Yamana, Ayana Uchimura, Tadaaki Inano, Tomoiku Takaku, Yasuharu Hamano, Miki Ando

**Affiliations:** ^1^ Department of Hematology, Juntendo University Graduate School of Medicine, Tokyo, Japan; ^2^ Department of Cardiovascular Biology and Medicine, Juntendo University Graduate School of Medicine, Tokyo, Japan

**Keywords:** protracted SARS-CoV-2, Long Covid, hematological malignancies, immunocompromised, anti-CD20 therapy, rituximab, Bruton kinase (BTK) inhibitors, COVID-19 PCR cycle threshold (Ct)

## Abstract

Persistent COVID-19 is a well recognized issue of concern in patients with hematological malignancies. Such patients are not only at risk of mortality due to the infection itself, but are also at risk of suboptimal malignancy-related outcomes because of delays and terminations of chemotherapy. We report two lymphoma patients with heavily pretreated persistent COVID-19 in which ensitrelvir brought about radical changes in the clinical course leading to rapid remissions. Patient 1 was on ibrutinib treatment for mantle cell lymphoma when he developed COVID-19 pneumonia which was severe and ongoing for 2 months despite therapy with molnupiravir, multiple courses of remdesivir, one course of sotrovimab, tocilizumab, and steroids. Patient 2 was administered R-CHOP therapy for diffuse large B-cell lymphoma when he developed COVID-19 which was ongoing for a month despite treatment with multiple courses of remdesivir and one course of sotrovimab. A 5-day administration of ensitrelvir promptly resolved the persistent COVID-19 accommodated by negative conversions of RT-qPCR tests in both patients within days. Ensitrelvir is a novel COVID-19 therapeutic that accelerates viral clearance through inhibition of the main protease of SARS-CoV-2, 3-chymotrypsin-like protease, which is vital for viral replication. Ensitrelvir is a promising treatment approach for immunocompromised lymphoma patients suffering from persisting and severe COVID-19.

## 1 Introduction

Life-threatening severe COVID-19 is becoming less frequent compared to the earlier days of the pandemic owing to development of vaccines, novel therapeutics, and evolution of the virus to attenuated variants. However, this does not apply to patients with hematological malignancies because the immune system is often impaired due to the underlying malignancy itself, and also because the effects of chemotherapy targeted towards the malignancy linger for long periods of time. Persistent COVID-19 is a well recognized issues of concern in patients with hematological malignancies including lymphoma (1, 2). In lymphoma patients, protracted COIVD-19 can not only be severe and life-threatening by itself, but also may lead to discontinuation of chemotherapy and thereby result in death due to lymphoma. Lee et al. reported that among 214 patients with lymphoid malignancies complicated with COVID-19, 102 patients were hospitalized, 32 patients experienced an extended hospitalization of over 21 days, and 19 of the discharged patients were later readmitted for recurrent respiratory symptoms. Of the 19 readmitted patients, 13 patients experienced delays in chemotherapy and 4 patients died from persistent COVID-19 (3). There are no fundamental solutions for persistent COVID-19 to date. Vaccinations also have limited effectiveness, and compared to 98% of healthy controls achieving seroconversion, only 15% of patients undergoing anti-CD20 therapy within one year and 23% of patients on Bruton kinase (BTK) inhibitors have been reported to show successful seroconversion (4). We report two lymphoma patients with heavily pretreated COVID-19 ongoing for months who promptly attained remissions within days after administration of ensitrelvir. Ensitrelvir is a novel COVID-19 therapeutic that accelerates viral clearance through a unique mechanism involving protease inhibition. From our observations, ensitrelvir is a promising treatment approach for lymphoma patients suffering from persistent COVID-19.

## 2 Case presentation

**Figure 1:**
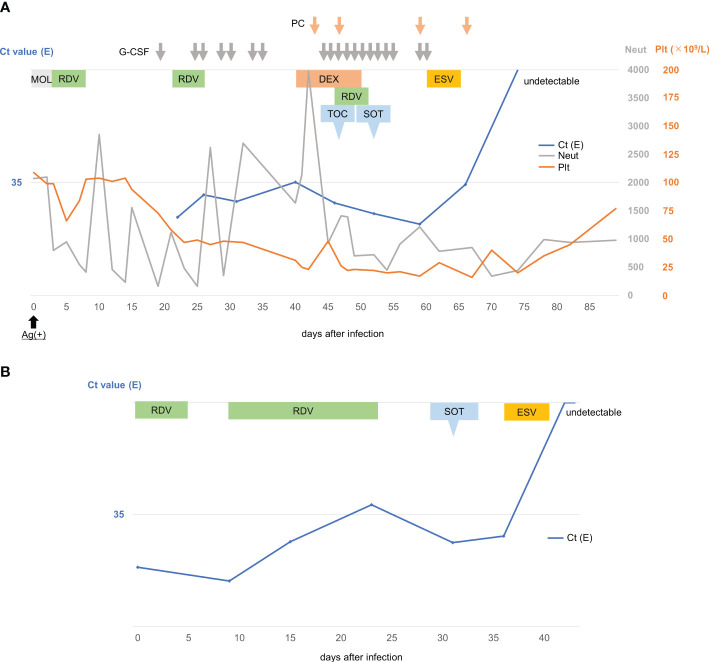
Clinical course of patients with persistent COVID-19 infections. **(A)**. Clinical course of a 76-year-old male with mantle cell lymphoma developing persistent COVID-19 infection. **(B)**. Clinical course of a 78-year-old male with diffuse large B-cell lymphoma developing persistent COVID-19 infection. Ag, antigen; Ct, cycle threshold; E, envelope; ESV, Ensitrelvir; DEX, dexamethasone; G-CSF, granulocyte-colony stimulating factor; MOL, Molnupiravir; Neut, neutrophil; PC, platelet concentrate; Plt, Platelet; RDV, Remdesivir; SOT, Sotrovimab; TOC, Tocilizumab.

Case 1 is a 76-year-old male patient with mantle cell lymphoma (MCL). Of previously reported risk factors for COVID-19 severity (5), he harbored factors such as old age, male sex, diabetes mellitus, and cerebral infarction. He had completed five COVID-19 vaccinations. In 2014, he received autologous stem cell transplantation for MCL and achieved a complete response. MCL relapsed in 2017, and he was administered bendamustine with rituximab therapy to which he showed no response. Subsequently, ibrutinib 420mg/day was initiated from October 2017 and brought about excellent disease control of MCL. Ibrutinib was continued, and in April 2023 he developed fever and tested positive on a SARS-CoV-2 antigen test (day 0). He was prescribed molnupiravir 1600mg/day and was initially treated as an outpatient, but was admitted to our institution on day 2 due to persistent fever and rapid decline of overall status. Chest X-rays revealed right lung pneumonia. Treatment was altered from molnupiravir to remdesivir (200mg on day 2, 100mg on days 3-6). Neutropenia (<500 neutrophils) and thrombocytopenia which were thought to be complications of SARS-CoV-2 viral infection were also seen at this time, and granulocyte colony-stimulating factor (G-CSF) and platelet transfusions were administered on days appropriate. Symptoms including fever resided, and the patient was discharged on day 15. However, he was readmitted on day 21 due to recurrent fever and a deteriorating general condition. COVID-19 reverse transcription quantitative polymerase Chain Reaction (RT-qPCR) test was positive with a cycle threshold (Ct) value for envelope gene (E) at 27.3, and he was judged to have persistent COVID-19. Although he required no oxygen, CT scans showed ongoing pneumonia. He was administered an additional 5-day course of remdesivir (200mg on day 22, 100mg on days 23-26). Fever resided transiently but recurred on day 30. Day 31 RT-qPCR testing showed little improvement with Ct value at 30.75 (E), but because the patient’s overall status improved, he was discharged on day 34. Fever persisted, and outpatient examination on day 40 revealed low blood oxygen levels and aggravation of reticular shadows on CT scans and he was again readmitted. RT-qPCR Ct value was 35.1 (E). Dexamethasone administration starting at 6 mg/day and tapering led only to transient improvement of oxygen demand, and remdesivir was again initiated (200mg on day 46, 100mg on days 47-50). However, on day 47, the oxygen demand exacerbated from 2L/min to 8L/min accompanied by aggravation of pneumonia on CT scans, and 8mg/kg of tocilizumab was coadministered. The pneumonia and oxygen demand rapidly improved, but day 52 RT-qPCR testing showed no improvement with Ct value at 28.0 (E), and sotrovimab 500mg was administered on day 52. Overall condition and oxygen demand improved to 0.5L/min, but day 59 RT-qPCR revealed an exacerbating Ct value of 25.7 (E). Chest CT scans displayed residual bilateral reticular shadows, and neutropenia and thrombocytopenia persisted. Ensitrelvir (375mg on day 60, 125mg on days 61-64) was administered, and day 66 RT-qPCR testing showed a trend of improvement with a Ct value of 34.5 (E), and day 73 RT-qPCR testing became negative for the first time (**Figure 1A**). Simultaneously, platelet and neutrophil counts also recovered which was thought to be the result of SARS-CoV-2 viral clearance, and there was no longer a need for G-CSF administration or platelet transfusions. The patient was discharged on day 84, remains well with a normalized physical condition, and COVID-19 and MCL have not recurred as of day 208.

Case 2 is a 78-year-old male with diffuse large B-cell lymphoma. Risk factors for COVID-19 severity included old age, male sex, hypertension, and cerebral infarction. He had completed five COVID-19 vaccinations. In May 2023, he underwent initial CHOP therapy at 80% dose, followed by rituximab therapy at 375mg/m^2^ four days later, and intrathecal chemotherapy (methotrexate 15mg, cytarabine 40mg, prednisolone 10mg) five days later. He developed fever and loss of appetite, became bedridden, and was diagnosed with COVID-19 by RT-qPCR testing with Ct value of 23.1 (E) 12 days after CHOP therapy (day 0). CT scans showed no signs of pneumonia. Remdesivir was administered for five days (200mg on day 0, 100mg on days 1-4), but day 9 RT-qPCR testing showed no improvement with Ct value of 20.14 (E). Immunoglobulin replacement therapy was administered on days 10-12. Remdesivir administration was resumed at 100mg/day along with COVID-19 RT-qPCR monitoring, and remdesivir was terminated after 15 consecutive days of administration because his Ct value rose to 37.19 (E) on day 23. However, on day 31, a follow up RT-qPCR test showed a decreased Ct value of 28.7 (E), and sotrovimab 500mg was administered on day 31. PCR testing on day 36 showed little improvement with Ct value of 30.16 (E), and ensitrelvir was initiated (375mg on day 36, 125mg on days 37-40). On days 42 and 43, for the first time, RT-qPCR tests resulted negative. His general condition improved, appetite recovered, and he became able to walk with support. The patient desired no further treatment, and he was discharged to a nursing home (**Figure 1B**).

## 3 Discussion

Ensitrelvir is the first oral noncovalent, nonpeptide inhibitor that suppresses SARS-CoV-2 replication. The drug targets the substrate binding pocket of the main protease of SARS-CoV-2, 3-chymotrypsin-like protease, which plays a crucial role in viral replication. Ensitrelvir is also known for its wide spectrum target, and has been reported to be effective towards five WHO-identified variants of concern including Alpha (B.1.1.7), Beta (B.1.351), Gamma (P.1), Delta (B.1.617.2), and Omicron (B.1.1.529) (6). In the general vaccinated population infected with mild to moderately severe COVID-19, ensitrelvir administration within 72 hours of symptom onset has been reported to be associated with shorter duration of symptoms, shorter duration of viral shedding, and less likeliness of developing abnormally lingering symptoms known as “long COVID” (7, 8). From our observations of the two presented patients, it is most probable that ensitrelvir is also effective for treating persistent COVD-19 in lymphoma patients. Cases 1 and 2 were both extremely heavily treated with COVID-19 therapeutics including remdesivir, sotrovimab, molnupiravir before ensitrelvir initiation on days 36 and 60 of infection, respectively. Although none of the previously administered therapeutics were sufficient for viral clearance, ensitrelvir promptly resolved the persistent COVID-19 and brought about undetectable RT-qPCR results for the first time in both patients. Furthermore, as for case 1, not only RT-qPCR positivity but protracted waxing and waning COVID-19 manifestations including fever, pneumonia, oxygen demand, neutropenia, and thrombocytopenia also promptly resolved after ensitrelvir administration on day 60. RT-PCR positivity in the general population has been reported to persist for a mean of 7.4 days when infected with COVID-19, and this data highlights the abnormal persistence of PCR positivity in the two lymphoma cases presented here. Although not as heavily pretreated as the two presented cases, there are two case reports of successful treatment with ensitrelvir in lymphoma patients experiencing persistent COVID-19. Sakamaki et al. reported a lymphoma patient with COVID-19 persisting for 7 weeks. The patient underwent first-line treatment with ensitrelvir, and Ct values rose from 22.0 to 41.0 along with significant decrease in CRP levels. Jung et al. reported a lymphoma patient with persistent COVID-19 refractory to an initial 5-day course of remdesivir, who was successfully treated with combination therapy of remdesivir and ensitrelvir starting on day 32 from onset. The patient showed negative RT-qPCR results on nasopharyngeal swabs on day 38.

Severity and Mortality of COVID-19 infections in lymphoma patients have been reported to be significantly higher compared to that of the general population, but on top of this, lymphoma patients often suffer from a state of persisting COVID-19 (9). Persistent COVID-19 is unique to the immunocompromised, and patients with hematological malignancies have been reported to fare the worst (1). Immunocompromised patients including lymphoma patients are often refractory to first-line treatment for COVID-19, and most often require sequential or combination therapy for viral eradication (10). Also, while the COVID-19 persists in such patients, SARS-CoV-2 has been reported to acquire multidrug-resistant mutations to antiviral and antibody therapy, which further complicates the situation (11). A literature review of 19 case reports including patients with lymphoma or chronic lymphocytic leukemia experiencing persistent COVID-19 (defined by ongoing COVID-19 symptoms or lung lesions) found that median persistence of COVID-19 infections was 65 days, and all 11 patients studied failed to develop anti-SARS-CoV-2 antibodies. 5 patients died, 1 patient was alive with ongoing COVID-19, and 13 patients eventually recovered from COVID-19 (12). Duléry et al. reported that out of 111 lymphoma patients admitted for COVID-19, 32 patients required prolonged in-hospital stay (defined as >30 days) with a median length of stay of 58 days. Only 2 out of 19 patients studied were found to develop anti-SARS-CoV-2 antibodies (13). Persisting COVID-19 in lymphoma patients have been reported to continue for as long as 12 months (2, 12). Lymphoma patients are not only threatened by COVID-19 itself, but depending on the type and status of lymphoma, the long delays of chemotherapy can also be life-threatening. One proposed strategy for treating persisting COVID-19 is convalescent plasma therapy. Hueso et al. reported 17 B-cell depleted patients of which 15 were lymphoma patients who experienced persisting COVID-19 continuing for a median of 56 days. All 17 patients failed to develop anti-SARS-CoV-2 antibodies, but convalescent plasma therapy achieved remissions in 16 patients within 48 hours (14). Betrains et al. reported on 5 lymphoma patients with previous rituximab therapy who developed persistent COVID-19. All 5 patients failed to develop anti-SARS-CoV-2 antibodies, but convalescent plasma therapy achieved remissions in 4 patients (15). Convalescent plasma therapy is a rational approach for B-cell depleted patients because anti-SARS-CoV-2 antibodies are key for viral clearance, and humoral immunity is mainly impaired in such patients. However, convalescent plasma is not easily accessible for most institutions, and a commercially available therapeutic like ensitrelvir would be much more realistic for treating persistent COVID-19. Immunocompromised patients other than lymphoma have also been reported to suffer from persistent COVID-19 that are refractory to many lines of therapy, and these patients may also benefit from treatment with ensitrelvir (10, 16). A drawback of ensitrelvir is that it has a long list of drugs that are contraindicated for coadministration, including widely used drugs such as olmesartan medoxomil, rivaroxaban, and simvastatin. However, most of these drugs are substitutable by other drugs with similar effects, and thus careful patient interrogation and drug planning beforehand can make ensitrelvir appliable to the majority of patients in need.

One limitation of this report is that we were only able to observe effectiveness of ensitrelvir in 2 lymphoma patients. However, both patients had persisting COVID-19 despite being extremely heavily treated for long periods of time, and first-time remissions were confirmed directly after ensitrelvir administration. Another limitation is that the RT-qPCR tests done in the two patients presented were a carried out on a mixture of nasal swabs and saliva, and the testing methodology was not consistent. Also, testing for type of SARS-CoV-2 variant was not carried out in the two patients, but Omicron variant infections accounted for the vast majority at the time in Japan. Ikeda et al. reported that prolonged viral shedding was seen in 36.9% of patients with hematological diseases infected with the Omicron variant, which was higher than the reported 13.9-25.4% in the pre-Omicron era, and therefore persistent COVID-19 may become a more troublesome issue in the Omicron era (17).

In conclusion, although our findings need to be confirmed in larger studies, ensitrelvir is a promising new treatment strategy for persistent COVID-19 in patients with hematological malignancies including lymphoma.

## Data availability statement

The raw data supporting the conclusions of this article will be made available by the authors, without undue reservation.

## Ethics statement

The studies were conducted in accordance with the local legislation and institutional requirements. The participants provided their written informed consent to participate in this study. Written informed consent was obtained from the individual(s) for the publication of any potentially identifiable images or data included in this article.

## Author contributions

CF: Writing – original draft, Investigation, Project administration. HY: Writing – original draft, Conceptualization, Methodology. MH: Conceptualization, Supervision, Writing – original draft. SS: Writing – review & editing, Data curation, Methodology. TY: Writing – review & editing, Data curation. AU: Writing – review & editing, Data curation. TI: Writing – review & editing, Data curation. TT: Writing – review & editing, Data curation. YH: Writing – review & editing, Data curation. MA: Writing – review & editing, Supervision, Validation.
